# Contrast Sensitivity After Combined MIGS and Cataract Surgery: A Comparison of Monofocal and Enhanced Monofocal Intraocular Lenses

**DOI:** 10.3390/jcm15145477

**Published:** 2026-07-13

**Authors:** Keigo Takagi, Yuri Fujino, Hinako Ohtani, Chisako Ida, Kana Murakami, Mizuki Iida, Yuto Yoshida, Kazunobu Sugihara, Masaki Tanito

**Affiliations:** Department of Ophthalmology, Faculty of Medicine, Shimane University, Izumo 693-8501, Shimane, Japan

**Keywords:** contrast sensitivity, MIGS, enhanced monofocal IOL, monofocal IOL, glaucoma

## Abstract

**Background/Objectives**: Contrast sensitivity (CS) is an important measure of visual function, particularly in patients with glaucoma. While minimally invasive glaucoma surgery (MIGS) combined with cataract surgery is increasingly performed, postoperative CS outcomes—especially comparing enhanced monofocal intraocular lenses (emIOLs) and monofocal intraocular lenses (mIOLs)—remain unclear. This study aimed to compare postoperative CS between emIOL and mIOL in eyes undergoing combined MIGS and cataract surgery. **Methods**: This retrospective observational study included eyes that underwent combined MIGS and cataract surgery at a single center. CS was measured preoperatively and at 2–3 months postoperatively using the imo vifa system and quantified as the area under the log contrast sensitivity function (AULCSF). Clinical parameters were compared between IOL types using univariate and multivariate analyses, including a generalized linear mixed model (GLMM). Propensity score matching was performed to adjust for baseline differences. **Results**: A total of 140 eyes (71 emIOL, 69 mIOL) were analyzed. CS significantly improved postoperatively in both groups (*p* < 0.001). In multivariate analysis, IOL type was not significantly associated with CS (*p* = 0.10), whereas postoperative status was associated with improved CS (*p* = 0.01). CS measurement pattern significantly affected results, but no interaction between IOL type and measurement pattern was observed. After propensity score matching, postoperative CS was higher in the emIOL group (*p* = 0.01); however, multivariate analysis still showed no significant association between IOL type and CS (*p* = 0.20). **Conclusions**: CS improved after combined MIGS and cataract surgery regardless of IOL type. No independent difference in CS was observed between emIOL and mIOL, suggesting that emIOL may provide comparable visual quality in terms of CS, even in glaucoma patients.

## 1. Introduction

Contrast sensitivity (CS) is an important indicator of visual function in daily life. After cataract surgery, improvements in CS have been shown to be associated with better visual function and improved quality of vision [1,2]. Cataract surgery improves not only visual acuity but also CS under both photopic and mesopic conditions. These improvements may contribute to better performance in daily activities and higher patient satisfaction [3]. Monofocal intraocular lenses (mIOL) are commonly used in cataract surgery. In recent years, enhanced monofocal IOLs (emIOL) with extended depth of focus have been developed through optimization of optical design and spherical aberration control [4]. EmIOLs are designed to improve intermediate vision while maintaining contrast characteristics comparable to those of conventional mIOLs, and reports on their clinical outcomes have increased in recent years [5,6,7,8]. In patients undergoing cataract surgery alone, postoperative CS has been reported to be comparable between emIOLs and mIOLs [5,9]. However, glaucoma is frequently associated with reduced CS despite relatively preserved visual acuity [10]. Reduced CS has been linked to impaired mobility, difficulties with night driving and other low-light visual tasks, reduced vision-related quality of life, and deterioration of functional vision in glaucoma patients [10,11,12,13]. Therefore, CS represents an important functional outcome when evaluating IOL performance in glaucoma patients.

Minimally invasive glaucoma surgery (MIGS) is a glaucoma surgical approach that can be performed without conjunctival incision [14]. Most MIGS procedures target the trabecular meshwork, such as goniotomy, and are frequently combined with cataract surgery [14]. In recent years, various MIGS devices, including the iStent (Glaukos Corporation, San Clemente, CA, USA), XEN (Allergan, Dublin, Ireland), STREAMLINE (New World Medical, Rancho Cucamonga, CA, USA), and Hydrus Microstent (Alcon Vision LLC, Fort Worth, TX, USA), have been developed [15,16,17,18]. Accordingly, the number of clinical reports evaluating outcomes after MIGS has increased [18]. However, postoperative CS has not been a primary focus of evaluation in previous studies of combined MIGS and cataract surgery. In addition, to the best of our knowledge, postoperative visual function outcomes of MIGS combined with emIOL have not been reported.

In this study, we primarily compared postoperative CS between emIOL and mIOL in patients undergoing combined MIGS and cataract surgery. We also evaluated changes in pre- and postoperative clinical parameters.

## 2. Materials and Methods

### 2.1. Subjects

This single-center retrospective observational study was conducted at Shimane University Hospital using existing clinical records from patients who underwent surgery between August 2023 and November 2025. Data extraction and statistical analyses were initiated only after approval by the Institutional Review Board of Shimane University Hospital (Approval No. 20260115-1), which approved the retrospective use of these clinical data. The study adhered to the tenets of the Declaration of Helsinki. Inclusion criteria were eyes that underwent combined MIGS and cataract surgery at our institution during the study period. Eligible cases were limited to those operated on by a single experienced surgeon (M.T.). MIGS procedures included ab interno trabeculotomy using the Tanito microhook (TMH), Kahook Dual Blade (KDB), or STREAMLINE, as well as trabecular bypass stent implantation with iStent inject W or Hydrus Microstent. Only eyes implanted with either an emIOL, Vivinex Impress^®^ (HOYA Surgical Optics, Tokyo, Japan), or an mIOL, Vivinex iSert^®^ (HOYA Surgical Optics), were eligible for inclusion. In addition, CS had to be measured using the imo vifa system (CREWT Medical Systems, Tokyo, Japan) both preoperatively and at 2–3 months postoperatively. Exclusion criteria were preoperative visual acuity of counting fingers or worse and a history of previous filtration surgery or tube shunt implantation. At our institution, emIOL implantation was introduced in October 2024. Therefore, all eligible emIOL cases identified after its introduction were included, and an approximately equal number of mIOL cases were retrospectively selected from surgeries performed during the study period to balance the number of eyes between IOL types. Consequently, 71 eyes from 43 patients were included in the emIOL group and 69 eyes from 39 patients in the mIOL group.

### 2.2. Measurements

Subject demographics included age, sex, laterality, glaucoma subtype, and surgical procedure. Surgical procedures were categorized as goniotomy-based or implant-based procedures because procedures within each category were considered broadly comparable for the present outcomes [19,20], whereas device-specific analyses would have resulted in small and highly unbalanced subgroups. Eye-based parameters included best-corrected visual acuity (BCVA), intraocular pressure (IOP) measured using Goldmann applanation tonometry, spherical equivalent refractive error (SERE), corneal endothelial cell density (CECD) measured with a specular microscope (EM-3000; Tomey, Nagoya, Japan), anterior chamber (AC) flare measured with a laser flare meter (FM-600; Kowa, Nagoya, Japan), glaucoma medication score, visual field mean deviation (MD) assessed using the 30–2 Standard program of the Humphrey Visual Field Analyzer (Carl Zeiss Meditec, Dublin, CA, USA), and axial length (AL) measured with an optical biometer (OA-2000; Tomey, Nagoya, Japan). Among the eye-based parameters, visual field MD and AL were recorded only preoperatively as baseline ocular characteristics. The remaining eye-based parameters were assessed both preoperatively and at 2–3 months postoperatively. The medication score was defined as the total number of active antiglaucoma agents used. Fixed-dose combination eye drops were counted based on the number of active components rather than as a single medication.

### 2.3. Measurements of Contrast Sensitivity

CS was measured using the imo vifa system. All measurements were performed under photopic conditions and conducted monocularly, with the fellow eye fully occluded during testing. CS was assessed using two stimulus patterns, ring and stripe patterns, in accordance with the standard protocol of the device. Measurements were obtained separately for each eye. The area under the log CS function (AULCSF) obtained by measurement was used for quantitative analysis.

### 2.4. Surgical Procedure

The detailed surgical procedures have been described in our previous reports [21,22]. Briefly, MIGS procedures were performed according to the selected technique in combination with cataract surgery.

For TMH or KDB or STREAMLINE goniotomy, the procedure was performed after completion of standard phacoemulsification and IOL implantation. A viscoelastic agent (ProVisc^®^, sodium hyaluronate 1.0%, Alcon Laboratories, Fort Worth, TX, USA) was injected to maintain the anterior chamber. In right eyes, a 2.2-mm clear corneal incision was created at the 9 o’clock position with a slit knife (MANI, Tochigi, Japan), and the surgical instrument was introduced through this incision. In left eyes, a 20-gauge microvitreoretinal knife (MANI) was used to create a corneal side port at the 2–3 o’clock position, through which the instrument was inserted. Using a Swan–Jacob gonioprism lens for angle visualization, the trabecular meshwork and the inner wall of Schlemm’s canal were incised over approximately 120 degrees on the nasal side for TMH or KDB. For STREAMLINE goniotomy, focal viscodilation of Schlemm’s canal was performed via multiple micro-punctures along the nasal angle. After that, an additional 90-degree trabeculotomy of the nasal trabecular meshwork was performed using the device tip.

For trabecular bypass stent implantation, iStent inject W or Hydrus Microstent was used. Hydrus Microstent implantation was performed prior to phacoemulsification, whereas iStent inject W implantation was performed after completion of cataract surgery. In eyes receiving iStent inject W, two stents were implanted into the nasal trabecular meshwork under gonioscopic visualization. The stents were placed with an interval corresponding to approximately two clock hours between them. In right eyes, the delivery system was inserted through a 2.2-mm clear corneal incision created at the 9 o’clock position. In left eyes, the device was inserted through a corneal side port created at the 2–3 o’clock position. For Hydrus Microstent implantation, an additional corneal side port was created at the 8 o’clock position in right eyes and at the 4 o’clock position in left eyes, and the device was placed into the inferonasal trabecular meshwork under gonioscopic visualization.

After completion of the MIGS procedure, the viscoelastic material was thoroughly removed using irrigation and aspiration, and the corneal incisions were sealed by stromal hydration. At the conclusion of surgery, a subconjunctival injection of 2 mg betamethasone sodium phosphate was administered, and 0.3% ofloxacin ointment was applied. Postoperatively, topical 1.5% levofloxacin and 0.1% betamethasone eye drops were prescribed four times daily for 3–4 weeks.

### 2.5. Statistical Analysis

Statistical analyses were performed to evaluate clinical factors associated with CS. Continuous variables are presented as mean ± standard deviation with 95% confidence intervals, and categorical variables are presented as counts and percentages. Comparisons of baseline continuous variables between IOL types were performed using the Student’s *t* test, whereas categorical variables were compared using Fisher’s exact test.

Preoperative and postoperative clinical parameters were analyzed as follows. At each time point, comparisons between IOL types were performed using the Student’s *t* test. Comparisons between preoperative and postoperative measurements were conducted separately within each IOL type using the paired t test; however, medication score, which did not follow a normal distribution, was analyzed using the Wilcoxon signed-rank test.

To identify factors associated with CS, a generalized linear mixed model (GLMM) assuming a Gaussian distribution with an identity link function was constructed. CS was treated as the dependent variable. Fixed effects included age, sex, glaucoma subtype, MIGS procedure, CS measurement pattern (ring/stripe), IOL type (em/m), time point (post/pre), BCVA, IOP, SERE, CECD, AC flare, visual field MD, AL, and glaucoma medication score. An interaction term between measurement pattern and IOL type was also included. This was because CS measurement patterns were not evenly distributed between IOL types, with emIOL predominantly measured using the stripe pattern and mIOL predominantly measured using the ring pattern. Patient ID was included as a random effect to account for inter-eye correlation. The patient IDs used for analysis were anonymized study-specific identifiers, and no directly identifiable patient information was included in the analytic dataset. One case had a missing CECD value, and four cases had missing AC flare values. These observations were excluded from the mixed-effects model by complete-case analysis. Multicollinearity among fixed effects was assessed using variance inflation factors (VIFs) calculated from an ordinary least squares regression model.

To further minimize confounding due to baseline differences between IOL groups, propensity score matching was performed. Propensity scores were calculated using a logistic regression model including preoperative BCVA, IOP, CS measurement pattern, visual field MD, and glaucoma medication score. One-to-one nearest neighbor matching without replacement was conducted using a caliper width of 0.2. Baseline characteristics and postoperative outcomes were re-evaluated using the matched dataset. Multivariate analysis after propensity score matching was performed using a GLMM including IOL type, time point, measurement pattern, and their interaction terms. Patient ID was included as a random effect to account for inter-eye correlation. No missing data were present in the variables used for propensity score matching.

All statistical analyses were performed using JMP Student Edition (version 19; SAS Institute Inc., Cary, NC, USA). A two-sided *p* value < 0.05 was considered statistically significant. Significance levels of 1% (*p* < 0.01) and 0.1% (*p* < 0.001) were also reported.

## 3. Results

Table 1 shows the preoperative baseline characteristics of patients, including subject demographics and eye-based parameters. Significant differences between two groups were observed in the performed MIGS procedure (*p* < 0.001), CS measurement pattern (*p* < 0.001), and visual field MD (*p* = 0.002). There were no significant differences in age, sex, axial length, and eye laterality.

Univariate analysis of clinical parameters evaluated both preoperatively and postoperatively is shown in Table 2. These analyses included comparisons between IOL groups, and preoperative and postoperative values within each IOL group. In comparisons between IOL groups, among preoperative parameters, the medication score was significantly higher in the mIOL group (*p* < 0.001), and among postoperative parameters, AC flare was significantly higher in the emIOL group (*p* < 0.001). In pre- and post-operative comparisons, eye-based parameters, including BCVA (*p* = 0.001 and *p* < 0.001), IOP (both *p* < 0.001), SERE (*p* = 0.002 and *p* < 0.001), CECD (both *p* < 0.001), AC flare (*p* = 0.004 and *p* < 0.001), medication score (both *p* < 0.001), and CS (both *p* < 0.001), showed significant postoperative changes in both IOL groups. Pre- and post-operative CS was significantly lower in the mIOL group (*p* < 0.001), and CS showed significant postoperative improvement in both IOL groups (*p* < 0.001).

Table 3 presents the multivariate analysis of factors associated with CS. In this model, the inclusion of both eyes and repeated measurements, as well as variations of background parameters were adjusted. As a result, difference in IOL groups was not significantly associated with CS (Estimate = 0.17, *p* = 0.10). In contrast, CS measurement pattern showed a significant negative association, with the ring pattern associated with lower CS compared with the stripe pattern (Est = −0.37, *p* = 0.001). However, no significant interaction was observed between CS measurement pattern and IOL type (Est = 0.04, *p* = 0.78), suggesting that IOL type itself was not an indicator of CS. Postoperative time point showed significant association with higher CS (*p* = 0.01), suggesting a postoperative CS improvement. Among other parameters, higher age, lower BCVA, higher IOP, and lower visual field MD showed a significant association with lower CS (Est = −0.01, *p* = 0.048; Est = −0.93, *p* < 0.001; and Est = −0.02, *p* = 0.001; and Est = 0.01, *p* = 0.001, respectively). All VIF values were below 3.0, indicating no evidence of problematic multicollinearity.

Propensity score matching was performed at the eye level using BCVA, IOP, CS measurement pattern, visual field MD, and medication score to balance baseline characteristics between the IOL groups. After matching, 46 eyes from 33 patients were included, comprising 23 eyes from 16 patients in the emIOL group and 23 eyes from 17 patients in the mIOL group. Preoperative baseline characteristics of the matched emIOL and mIOL groups are summarized in Table 4. After propensity score matching, no significant differences in baseline characteristics were observed between the IOL groups.

Table 5 summarizes the comparisons of preoperative and postoperative clinical parameters between matched emIOL and mIOL groups. These analyses included comparisons between IOL groups as well as pre- and post-operative comparisons within each IOL group. Compared with the mIOL group, postoperative CS was significantly higher in the emIOL group (*p* = 0.013). Additionally, postoperative IOP, AC flare, and medication score were significantly higher in the emIOL group than the mIOL group (*p* = 0.02, *p* < 0.001, and *p* = 0.04, respectively). Compared with preoperative parameters, all parameters showed significant changes postoperatively in both the emIOL and mIOL groups, including BCVA (*p* = 0.002 and *p* = 0.008), IOP (*p* = 0.009 and *p* < 0.001), SERE (*p* = 0.004 and *p* = 0.02), CECD (*p* = 0.007 and *p* = 0.004), AC flare (*p* < 0.001 and *p* = 0.003), medication score (*p* < 0.001 and *p* < 0.001), and CS (*p* < 0.001 and *p* = 0.004).

Table 6 presents the multivariate analysis of CS after propensity score matching. Given that preoperative parameters were equivalent between IOL groups after matching, CS measurement pattern, IOL type, time point (postoperative vs. preoperative), and the interaction between CS measurement pattern and IOL type were included as covariates. Even after matching, difference in IOL type was not significantly associated with CS (Est = 0.04, *p* = 0.79). In this model, CS measurement pattern demonstrated a significant negative association, with ring patterns associated with lower CS compared with stripe patterns (Est = −0.39, *p* = 0.02). However, no significant interaction was observed between CS measurement pattern and IOL type (Est = 0.13, *p* = 0.54) suggesting that difference in IOL type was not associated with CS. Postoperative time point showed a significant association with higher CS (Est = 0.40, *p* < 0.001).

## 4. Discussion

This retrospective study investigated postoperative CS in eyes undergoing combined MIGS and cataract surgery, comparing emIOL with mIOL. Postoperative CS improved significantly from preoperative levels in both IOL groups. In the univariable analyses, postoperative CS was significantly higher in the emIOL group both before and after propensity score matching. However, multivariable analyses consistently demonstrated no significant association between IOL type and CS. These findings suggest that emIOL was not independently associated with postoperative CS in eyes undergoing combined MIGS and cataract surgery.

In this study, CS did not differ between both em and mIOL groups in combined MIGS and cataract surgery. Several previous studies have compared emIOLs and mIOLs in cataract surgery alone. Previous studies comparing emIOL with mIOL have reported no significant differences in CS under mesopic conditions [23,24]. Other reports comparing three types of emIOLs (Tecnis Eyhance, Nidek NSP-3, Hoya Vivinex Impress) with a mIOL (Tecnis ZCB00V) have shown no differences in contrast visual acuity under either photopic or mesopic conditions [25]. Their findings are consistent with our results. These findings suggest that the use of emIOL in combined MIGS and cataract surgery provides postoperative CS improvements comparable to those achieved with mIOL, even in glaucoma patients, a population in whom CS is often compromised.

A potential source of bias in this study was the change in the CS measurement pattern following a software update of the imo vifa system in September 2024. This transition partially overlapped with the introduction of emIOL implantation, resulting in an imbalance between IOL type and CS measurement pattern across the study cohort. To account for this temporal overlap, we included an interaction term between IOL type and measurement pattern in the multivariable analysis. The measurement pattern itself was significantly associated with CS, demonstrating a difference between the ring and stripe patterns. However, no significant interaction between IOL type and measurement pattern was observed, and no significant difference in CS was detected between IOL types. In multivariate analysis performed after propensity score matching, the association between measurement pattern and CS remained significant. In contrast, no significant difference in CS was found between IOL types. Therefore, the overall conclusion of this study remains unchanged.

Postoperative CS has been reported to show relatively limited variability, with standard deviations generally around 0.2 in routine cataract surgery populations [26,27]. In contrast, the present study showed greater variability in AULCSF (SD approximately 0.4–0.5). This may be explained by the inclusion of glaucoma patients undergoing combined MIGS and cataract surgery, a population characterized by heterogeneous disease severity and visual function. Test–retest repeatability studies of the AULCSF metric have demonstrated coefficients of repeatability for AULCSF in the range of approximately 0.17–0.39 log units, indicating substantial measurement variability under typical clinical testing conditions [28]. Under these assumptions, and based on conventional power considerations used in prior CS studies, a comparison between approximately 70 eyes per group provides an expected statistical power of around 0.8 to detect such a difference. These considerations suggest that the sample size was unlikely to be the sole explanation for the absence of a significant difference between IOL groups.

EmIOLs have been increasingly adopted in cataract surgery because they provide improved intermediate vision while maintaining visual quality comparable to that of mIOLs [5,29]. In addition, their use in patients with glaucoma has been increasingly reported [30,31,32]. The appropriate use of premium IOLs, including toric designs, in glaucoma patients has become an important and evolving topic in contemporary cataract surgery. In the present study, we specifically focused on postoperative CS in eyes undergoing combined MIGS and cataract surgery. However, intermediate and near visual acuity were not evaluated and should be considered a limitation of this study. Mesopic contrast sensitivity, glare, halos, pupil size, and ocular aberrations were also not assessed because these data were not routinely collected in clinical practice.

In addition to the limited assessment of distance-specific visual acuity, this study has several limitations. First, the retrospective single-center design and non-randomized IOL selection may have introduced selection bias. Thus, we attempted to mitigate this bias through propensity score matching and multivariable analysis. Nevertheless, in the matched postoperative comparison, significant differences were observed in IOP, AC flare, medication score, and CS. Although postoperative CS remained significantly higher in the emIOL group in the univariable analysis after propensity score matching, this association was not retained in the multivariable analysis. Therefore, the observed differences, including those in CS, may reflect residual confounding related to patient characteristics, postoperative conditions, or measurement patterns rather than true effects of IOL type. Because the emIOL and mIOL share the same material and basic lens design platform, these residual differences are more likely to reflect unmeasured confounding than genuine IOL-related effects. Second, CS was assessed using different measurement patterns. Although pattern effects and interactions with IOL type were statistically addressed, this methodological variability may have contributed to the large dispersion of AULCSF observed in this glaucoma population. In addition, a post-update-only validation analysis was not performed because the subgroup assessed after the software update was small and unbalanced between IOL groups. Third, a one-eye-per-patient sensitivity analysis was not performed because excluding one eye from bilateral cases would have further reduced the already limited sample size. Accordingly, the possible influence of patient-level clustering due to the inclusion of both eyes from some patients cannot be completely excluded. Fourth, postoperative follow-up was relatively short and may be insufficient to evaluate long-term IOP changes or visual field progression. However, prior cataract-only studies have evaluated visual outcomes at 2 months [33], and BCVA after MIGS has been reported to stabilize within 2–6 months [34,35]. Therefore, it was adequate to assess early postoperative functional outcomes, including CS.

Future studies should move beyond simply determining whether emIOLs preserve CS after combined MIGS and cataract surgery and should instead clarify how emIOLs contribute to postoperative visual rehabilitation in glaucoma eyes. Because postoperative visual quality after combined surgery may be influenced not only by IOL optics but also by IOP reduction, medication reduction, ocular surface status, postoperative inflammation, and glaucoma severity, future prospective studies should evaluate these factors together. Such an approach may help identify which glaucoma patients are most likely to benefit from emIOL implantation and define a more individualized strategy for IOL selection in eyes undergoing MIGS.

## 5. Conclusions

In conclusion, CS improved after combined MIGS and cataract surgery, and no independent difference was observed between emIOL and mIOL. These findings suggest that postoperative CS outcomes may be comparable between emIOL and mIOL in glaucoma patients undergoing MIGS. Further prospective studies are needed to confirm these findings.

## Figures and Tables

**Table 1:** Preoperative baseline characteristics.

	emIOL	mIOL	*p*-Value
N, subjects	43	39	
Age, years			
	71.9 ± 7.9	70.5 ± 9.3	0.35
	70.0, 73.8	68.3, 72.8	
Sex			
male	21 (49%)	15 (38%)	0.34
female	22 (51%)	24 (62%)	
N, eyes	71	69	
Laterality			
right	34 (48%)	34 (49%)	1.0
left	37 (52%)	35 (51%)	
Glaucoma Subtype			
POAG	54 (76%)	54 (78%)	0.39
EXG	13 (18%)	8 (12%)	
Other	4 (6%)	7 (10%)	
MIGS procedure			
goniotomy	17 (24%)	46 (67%)	<0.001 ***
implant	54 (76%)	23 (33%)	
Axial length, mm			
	24.5 ± 1.9	25.0 ± 1.8	0.13
	24.0, 24.9	24.5, 25.4	
Visual field MD, dB			
	−9.1 ± 7.4	−13.3 ± 8.2	0.002 **
	−10.9, −7.3	−15.3, −11.3	
CS measurement pattern			
ring	14 (20%)	57 (83%)	<0.001 ***
stripe	57 (80%)	12 (17%)	

Data are expressed in mean ± standard deviation for continuous variables, and in N (%) for categorical variables. *p*-values are estimated using Student’s *t* test for continuous variables and Fisher’s exact test for categorical variables. MIGS procedures are classified into goniotomy and implant-based procedures. Goniotomy includes ab-interno trabeculotomy using a Tanito microhook or Kahook Dual Blade, and STREAMLINE. Implant-based procedures included trabecular bypass stents, specifically the iStent inject W and the Hydrus Microstent. The **, and *** indicate significance levels at 1% (*p* < 0.01), and 0.1% (*p* < 0.001), respectively. IOL, intraocular lens; emIOL, enhanced monofocal IOL; mIOL, monofocal IOL; SD, standard deviation; CI, confidence interval; POAG, primary open-angle glaucoma; EXG, exfoliation glaucoma; MIGS, minimally invasive glaucoma surgery; MD, mean deviation; CS, contrast sensitivity.

**Table 2:** Comparison of pre- and post-operative clinical parameters between emIOL and mIOL groups by univariate analysis.

	emIOL	mIOL	*p*-Value ^a^
BCVA, logMAR			
Pre	0.2 ± 0.3 (0.1, 0.3)	0.2 ± 0.3 (0.1, 0.2)	0.59
Post	0.1 ± 0.2 (0.0, 0.1)	0.1 ± 0.3 (0.0, 0.1)	0.89
*p*-value ^b^	0.001 **	<0.001 ***	
IOP, mmHg			
Pre	16.6 ± 5.5 (15.3, 17.9)	16.8 ± 4.9 (15.6, 18.0)	0.83
Post	12.9 ± 3.1 (12.2, 13.6)	13.2 ± 2.7 (12.5, 13.8)	0.56
*p*-value ^b^	<0.001 ***	<0.001 ***	
SERE, D			
Pre	−2.1 ± 4.5 (−3.2, −1.0)	−3.5 ± 3.8 (−4.4, −2.5)	0.05
Post	−0.5 ± 1.1 (−0.8, −0.3)	−0.9 ± 1.5 (−1.3, −0.6)	0.07
*p*-value ^b^	0.002 **	<0.001 ***	
CECD, cells/mm^2^			
Pre	2465 ± 349 (2383, 2548)	2419 ± 264 (2356, 2483)	0.38
Post	2143 ± 640 (1990, 2295)	2240 ± 355 (2155, 2326)	0.27
*p*-value ^b^	<0.001 ***	<0.001 ***	
AC flare, pc/ms			
Pre	10.8 ± 7.6 (9.0, 12.6)	10.0 ± 5.5 (8.6, 11.4)	0.48
Post	29.4 ± 54.7 (16.3, 42.6)	16.0 ± 9.0 (13.8, 18.1)	0.049 *
*p*-value ^b^	0.004 **	<0.001 ***	
Medication score			
Pre	2.6 ± 1.3 (2.3, 3.0)	3.5 ± 1.2 (3.2, 3.8)	<0.001 ***
Post	1.5 ± 0.9 (1.3, 1.7)	1.6 ± 0.8 (1.4, 1.8)	0.44
*p*-value ^b^	<0.001 ***	<0.001 ***	
CS, AULCSF			
Pre	1.4 ± 0.5 (1.3, 1.6)	0.9 ± 0.4 (0.8, 1.0)	<0.001 ***
Post	1.7 ± 0.4 (1.6, 1.8)	1.3 ± 0.5 (1.1, 1.4)	<0.001 ***
*p*-value ^b^	<0.001 ***	<0.001 ***	

Data are expressed in mean ± SD (95% CI). ^a^ *p*-value for comparisons between IOL types for pre- or post-operative parameters using the Student’s *t* test. ^b^ *p*-Value for comparisons between pre- and post-operative values using the paired *t* test, except for medication score, which was analyzed using the Wilcoxon signed-rank test. The *, **, and *** indicate significance levels at 5% (*p* < 0.05) 1% (*p* < 0.01), and 0.1% (*p* < 0.001), respectively. SD, standard deviation; CI, confidence interval; IOL, intraocular lens; emIOL, enhanced monofocal IOL; mIOL, monofocal IOL; BCVA, best-corrected visual acuity; LogMAR, Logarithm of the Minimum Angle of Resolution; IOP, intraocular pressure; SERE, spherical equivalent refractive error; CECD, corneal endothelial cell density; AC, anterior chamber; CS, contrast sensitivity; AULCSF, area under the log contrast sensitivity function.

**Table 3:** Multivariate analysis of factors associated with CS.

Parameters	Estimate	95% CI	*p*-Value
Age, /years	−0.01	−0.01, 0.00	0.048 *
Sex (female/male)	0.01	−0.11, 0.12	0.90
Glaucoma subtype			
EXG/POAG	−0.06	−0.22, 0.10	0.48
Other/POAG	−0.11	−0.31, 0.09	0.29
MIGS procedure (implant/goniotomy)	−0.01	−0.14, 0.12	0.84
BCVA, /logMAR	−0.93	−1.11, −0.75	<0.001 ***
IOP, /mmHg	−0.02	−0.03, −0.01	0.001 **
SERE, /D	0.01	−0.01, 0.03	0.27
Axial Length, /mm	−0.03	−0.07, 0.00	0.07
CECD, /cells/mm^2^	0.00	0.00, 0.00	0.59
AC flare, /pc/ms	0.00	0.00, 0.00	0.31
Visual field MD, /dB	0.01	0.00, 0.02	0.001 **
Medication score	0.03	−0.01, 0.07	0.19
CS measurement pattern (ring/stripe)	−0.37	−0.59, −0.15	0.001 **
IOL type (enhanced/mono)	0.17	−0.03, 0.37	0.10
CS measurement pattern × IOL type	0.04	−0.24, 0.33	0.78
Time (post/pre)	0.14	0.03, 0.26	0.01 *

*p*-values are estimated using a Gaussian generalized linear mixed model with an identity link, including patient ID as a random effect to account for repeated measurements and inter-eye correlation. The *, **, and *** indicate significance levels at 5% (*p* < 0.05) 1% (*p* < 0.01), and 0.1% (*p* < 0.001) respectively. MIGS procedures are classified into goniotomy and implant-based procedures. Goniotomy included ab-interno trabeculotomy using a Tanito microhook or Kahook Dual Blade, and STREAMLINE. Implant-based procedures included trabecular bypass stents, specifically the iStent inject W and the Hydrus Microstent. CI, confidence interval; EXG, exfoliation glaucoma; POAG, primary open-angle glaucoma; MIGS, minimally invasive glaucoma surgery; IOL, intraocular lens; BCVA, best-corrected visual acuity; LogMAR, Logarithm of the Minimum Angle of Resolution; IOP, intraocular pressure; SERE, spherical equivalent refractive error CECD, corneal endothelial cell density; AC, anterior chamber; MD, mean deviation; CS, contrast sensitivity.

**Table 4:** Preoperative baseline characteristics of propensity score matched emIOL and mIOL groups.

	emIOL	mIOL	*p*-Value
N, subjects	16	17	
Age, years			
	70.3 ± 8.3	70.5 ± 2.1	0.88
	66.7, 73.9	66.3, 74.8	
Sex			
male	7(44%)	8 (47%)	0.73
female	9 (56%)	9 (53%)	
N, eyes	23	23	
Laterality			
right	12 (52%)	8 (35%)	0.37
left	11 (48%)	15 (65%)	
Glaucoma Subtype			
POAG	18 (78%)	21 (91%)	0.19
EXG	5 (22%)	1 (4%)	
Other	0 (0%)	1 (4%)	
MIGS procedure			
goniotomy	7 (30%)	8(35%)	1.0
implant	16 (70%)	15 (65%)	
Axial length, mm			
	25.2 ± 2.2	25.2 ± 1.5	0.96
	24.2, 26.2	24.5, 25.8	
Visual field MD, dB			
	−11.3 ± 7.4	−10.3 ± 8.6	0.69
	−14.5, −8.1	−14.1, −6.6	
CS measurement pattern			
ring	12 (52%)	13 (57%)	1.0
stripe	11 (48%)	10 (43%)	

Data are expressed in mean ± standard deviation for continuous variables, and in N (%) for categorical variables. *p*-values are estimated using Student’s *t* test for continuous variables and Fisher’s exact test for categorical variables. MIGS procedures are classified into goniotomy and implant-based procedures. Goniotomy included ab-interno trabeculotomy using a Tanito microhook or Kahook Dual Blade, and STREAMLINE. Implant-based procedures included trabecular bypass stents, specifically the iStent inject W and the Hydrus Microstent. IOL, intraocular lens; emIOL, enhanced monofocal IOL; mIOL, monofocal IOL; SD, standard deviation; CI, confidence interval; POAG, primary open-angle glaucoma; EXG, exfoliation glaucoma; MIGS, minimally invasive glaucoma surgery; MD, mean deviation; CS, contrast sensitivity.

**Table 5:** Comparison of pre- and postoperative clinical parameters between propensity score matched emIOL and mIOL groups by univariate analysis.

	emIOL	mIOL	*p*-Value ^a^
BCVA, logMAR			
Pre	0.2 ± 0.2 (0.1, 0.3)	0.2 ± 0.4 (0.1, 0.4)	0.78
Post	0.0 ± 0.1 (0.0, 0.1)	0.1 ± 0.3 (0.0, 0.3)	0.20
*p*-value ^b^	0.002 **	0.008 **	
IOP, mmHg			
Pre	16.1 ± 5.2 (13.9, 18.3)	16.0 ± 3.9 (14.3, 17.7)	0.95
Post	14.1 ± 3.7 (12.4, 15.7)	11.8 ± 2.3 (10.8, 12.8)	0.02 *
*p*-value ^b^	0.009 **	<0.001 ***	
SERE, D			
Pre	−4.2 ± 5.7 (−6.6, −1.7)	−3.7 ± 4.2 (−5.5, −1.9)	0.76
Post	−1.0 ± 1.5 (−1.7, 0.3)	−1.7 ± 1.7 (−2.4, −1.0)	0.14
*p*-value ^b^	0.004 **	0.02 *	
CECD, cells/mm^2^			
Pre	2470 ± 283 (2348, 2593)	2436 ± 295 (2308, 2563)	0.69
Post	2207 ± 526 (1980, 2435)	2253 ± 303 (2122, 2384)	0.72
*p*-value ^b^	0.007 **	0.004 **	
AC flare, pc/ms			
Pre	12.4 ± 11.3 (7.5, 17.3)	8.4 ± 2.9 (7.1, 9.8)	0.13
Post	23.2 ± 9.5 (18.9, 27.6)	14.2 ± 6.3 (11.5, 17.0)	<0.001 ***
*p*-value ^b^	<0.001 ***	0.003 **	
Medication score			
Pre	3.0 ± 1.1 (2.6, 3.5)	3.0 ± 1.5 (2.3, 3.6)	0.82
Post	1.8 ± 0.8 (1.5, 2.2)	1.3 ± 0.9 (0.9, 1.7)	0.04 *
*p*-value ^b^	<0.001 ***	<0.001 ***	
CS, AULCSF			
Pre	1.2 ± 0.4 (1.0, 1.4)	1.1 ± 0.5 (0.9, 1.4)	0.73
Post	1.8 ± 0.3 (1.7, 1.9)	1.5 ± 0.5 (1.2, 1.7)	0.01 *
*p*-value ^b^	<0.001 ***	0.004 **	

Data are expressed in mean ± SD (95% CI). ^a^ *p*-value for comparisons between IOL types for pre- or post-operative parameters using the Student’s *t* test. ^b^ *p*-value for comparisons between pre- and post-operative values using the paired t test, except for medication score, which was analyzed using the Wilcoxon signed-rank test. The *, **, and *** indicate significance levels at 5% (*p* < 0.05) 1% (*p* < 0.01), and 0.1% (*p* < 0.001) respectively. SD, standard deviation; CI, confidence interval; IOL, intraocular lens; emIOL, enhanced monofocal IOL; mIOL, monofocal IOL; BCVA, best-corrected visual acuity; LogMAR, Logarithm of the Minimum Angle of Resolution; IOP, intraocular pressure; SERE, spherical equivalent refractive error; CECD, corneal endothelial cell density; AC, anterior chamber; CS, contrast sensitivity; AULCSF, area under the log contrast sensitivity function.

**Table 6:** Multivariate analysis of CS after propensity score matching.

	Estimate	95% CI	*p*-Value
CS measurement Pattern (ring/stripe)	−0.39	−0.72, −0.05	0.02 *
IOL type (enhanced/mono)	0.04	−0.28, 0.36	0.79
CS measurement pattern × IOL type	0.13	−0.30, 0.57	0.54
Time (post/pre)	0.40	0.25, 0.55	<0.001 ***

*p*-values are estimated using a Gaussian generalized linear mixed model with an identity link, including patient ID as a random effect to account for repeated measurements and inter-eye correlation. The * and *** indicate significance levels at 5% (*p* < 0.05) and 0.1% (*p* < 0.001) respectively. CI, confidence interval; CS, contrast sensitivity; IOL, intraocular lens.

## Data Availability

Data are fully available upon reasonable request to the corresponding author.

## Abbreviations

The following abbreviations are used in this manuscript:

AC	Anterior chamber
AL	Axial length
AULCSF	Area under the log contrast sensitivity function
BCVA	Best corrected visual acuity
CECD	Corneal endothelial cell density
CI	Confidence interval
CS	Contrast sensitivity
emIOL	Enhanced monofocal intraocular lens
EXG	Exfoliation glaucoma
GLMM	Generalized linear mixed model
IOP	Intraocular pressure
IOL	Intraocular lens
KDB	Kahook dual blade
LogMAR	Logarithm of the Minimum Angle of Resolution
MD	Mean deviation
MIGS	Minimally invasive glaucoma surgery
mIOL	Monofocal intraocular lens
POAG	Primary open angle glaucoma
SD	Standard deviation
SERE	Spherical equivalent refractive error
TMH	Tanito micro hook
